# Electroacupuncture Combined With Diet Treatment Has a Therapeutic Effect on Perimenopausal Patients With Abdominal Obesity by Improving the Community Structure of Intestinal Flora

**DOI:** 10.3389/fphys.2021.708588

**Published:** 2021-11-25

**Authors:** Jili Sheng, Geyao Yang, Xiaoqing Jin, Caijuan Si, Yuan’an Huang, Zhouxiao Luo, Tao Liu, Jianfang Zhu

**Affiliations:** ^1^Acupuncture Department, Zhejiang Hospital, Hangzhou, China; ^2^Acupuncture and Massage Department, Hangzhou Geriatric Hospital, Hangzhou, China; ^3^Nutritional Department, Zhejiang Hospital, Hangzhou, China; ^4^Massage Department, Zhejiang Hospital, Hangzhou, China; ^5^Acupuncture Department, Tonglu TCM Hospital, Hangzhou, China

**Keywords:** abdominal obesity, perimenopausal syndrome, 16S rRNA, intestinal flora, electroacupuncture

## Abstract

**Background:** This study explored the influences of electroacupuncture combined with dietary intervention on the intestinal flora in perimenopausal patients with abdominal obesity by using the 16s rRNA sequencing technology.

**Methods:** Perimenopausal patients with abdominal obesity were divided into the Electroacupuncture group and the Control group. Patients in the Control group received healthy lifestyle education, while those in the Electroacupuncture group received electroacupuncture combined with dietary intervention. Before and after treatment, the weight, height, waist circumference, hip circumference, waist-height ratio (WHtR), waist to hip ratio (WHR), and body mass index (BMI) of the patients were recorded; the levels of serum triglyceride (TG), total cholesterol (TC), low-density lipoprotein (LDL), high-density lipoprotein cholesterol (HDL-C), fasting insulin (FINS), and fasting blood glucose (FGB) were evaluated; and the abundance, diversity, and species differences of intestinal flora were analyzed using 16s rRNA sequencing technology.

**Results:** The body weight, waist circumference, hip circumference, BMI, WHR, and WHtR of patients in the Electroacupuncture group after treatment were lower than those before treatment. Compared with the Control group, patients in the Electroacupuncture group after treatment displayed lower waist circumference, WHtR, WHR, TG, and LDL levels as well as species abundance, higher species diversity, and lager species difference in the intestinal flora. Besides, the proportions of Klebsiella and Kosakonia in the intestinal flora of patients in the Electroacupuncture group after treatment were larger than those before treatment.

**Conclusion:** Electroacupuncture combined with diet treatment generated a therapeutic effect on abdominal obesity in perimenopausal patients by improving the community structure of intestinal flora.

## Introduction

With the improvement of people’s living standards and the changes in dietary structure and environment, the number of obese people has maintained an upward trend (Vandevijvere et al., 2015; González-Muniesa et al., 2017; Mohammadbeigi et al., 2018). Obesity induced by the excessive accumulation of adipose tissue in the abdominal cavity and around the mesentery is defined as abdominal obesity, also termed central obesity (Smith, 2015). Perimenopausal women are prone to suffer from abdominal obesity due to decreased ovarian function, slowed metabolism, reduced exercise, osteoporosis, and overnutrition (Keller et al., 2010; Łokieć et al., 2016). In addition, abdominal obesity may further lead to hypertension, hyperlipidemia, coronary heart disease, type 2 diabetes, cholecystitis, cholelithiasis, and rectal cancer, thereby seriously threatening human health (Smith, 2015; Hu et al., 2017).

The routine treatments for obesity normally are dietary intervention and hypoglycemic drugs, which also provide certain references for perimenopausal women with abdominal obesity (Mastorakos et al., 2010; Bronczyk-Puzon et al., 2015; Ruban et al., 2019). In the treatment of obese patients, pharmacotherapy cannot usually be used alone or as a first-line method (Samat et al., 2008; Tauqeer et al., 2018). In comparison, dietary intervention has proved to be a healthy and effective way to lose weight, but it needs long-term persistence. Accumulating studies have indicated that electroacupuncture can treat abdominal obesity safely and effectively, mainly by stimulating the body’s meridians and acupoints, improving body condition as a whole, regulating intake and enhancing body metabolism, so as to achieve the purpose of weight loss and waist thinning (Wang et al., 2008; Lei et al., 2017; Chen et al., 2018; Luo et al., 2018; Huang et al., 2019; Zhang et al., 2019). However, the effect mechanism of electroacupuncture on abdominal obesity in perimenopausal women is still dearth of in-depth exploration.

The prevalence of abdominal obesity has raised people’s curiosity of finding the balance factors that affect the occurrence and development of this disease (González-Muniesa et al., 2017). The relationship between intestinal flora and abdominal obesity has gradually become a research hotspot with increasing attention from scholars at home and abroad (Malamut, 2007; Gao X. et al., 2018). The human intestinal flora is a complex ecosystem constituted by bacteria, archaea, yeasts, and fibrous fungi that reside in the intestinal tract (Gao Y. et al., 2018). It is estimated that the adult gastrointestinal tract contains at least 1,000 different microorganisms with a total number of more than 100 trillion, 10 times more than the total number of human cells, and the stability, diversity, and quantity of the intestinal flora vary in different races (Ma and Wu, 2017; Sebastián Domingo and Sánchez Sánchez, 2018). Changes in the diversity and composition of the intestinal flora are closely related to diseases including obesity and behavioral disorders. Studies also proved that drug treatment of obesity can be realized by improving intestinal flora dysfunction (Li et al., 2020); however, whether the effect of electroacupuncture on abdominal obesity is associated with the regulation of flora dysfunction is unknown.

Considering that the 16s rRNA high-throughput sequencing technology is the main and cutting-edge method for microorganism analysis (Gao X. et al., 2018), we, in this study, attempted to explore the effect of electroacupuncture combined with dietary intervention on the intestinal flora in perimenopausal patients with abdominal obesity. Anthropometric measures are simple, non-invasive tools that can be used for the diagnosis and assessment of obesity (Gazarova et al., 2019). Currently, the most widely used indexes are body mass index (BMI), waist circumference (WC), waist-to-hip ratio (WHR), waist-height ratio (WHtR), etc. (Gazarova et al., 2019). In addition, obesity can easily cause metabolic disorders such as glucose and lipids (Jablonowska-Lietz et al., 2017). Therefore, the relevant serum indexes that are also pivotal for assessing obesity are tested, including serum triglyceride (TG), total cholesterol (TC), low-density lipoprotein (LDL), high-density lipoprotein cholesterol (HDL-C), and insulin resistance (HOMA-IR) (Jablonowska-Lietz et al., 2017). In this study, we also tried to explore the effect of electroacupuncture combined with dietary intervention on the intestinal flora in perimenopausal patients with abdominal obesity by studying the changes in the above-mentioned related indicators.

## Materials and Methods

### Ethics Statement

The study has been approved by the Ethics Committee of Zhejiang Hospital [No. 2018 (9K)] and all patients signed the written informed consent.

### Patient Population

A total of 73 perimenopausal patients with abdominal obesity who received treatment in the Acupuncture Department of Zhejiang Hospital from December 2017 to January 2019 were recruited in this study. The diagnostic criteria for perimenopausal period were formulated according to the chapter of “Menopausal Syndrome” in the 8th edition of “Obstetrics and Gynecology,” combined with “Chinese Obstetrics and Gynecology.” The details are as follows: (1) 40–60 years old; (2) Clinical manifestations of menstrual disorders for more than 3 months, or amenorrhea within a year, etc.; (3) One or more of the following possible related symptoms, such as typical symptoms of unstable vasomotor function, psychoneurological symptoms (anxiety, tension, depression, irritability, etc.), physical symptoms (dry skin, muscle, and bone pain, etc.) and symptoms of urogenital atrophy (vaginal dryness, pain during intercourse, leakage of urine, repeated urinary tract infections, etc.); (4) Imaging examination to exclude organic diseases such as uterus and ovaries. The standards for abdominal obesity were formulated with reference to the “Guidelines for the Prevention and Control of Overweight and Obesity in Chinese Adults”: waist circumference ≥ 80 cm, and (or) WHtR ≥ 0.50, and (or) WHR ≥ 0.78. The general information of the patients including age and height was recorded (Table 1).

**TABLE 1:** Comparison of age and height between the two groups of patients.

**Groups**	**Cases**	**Age (year)**	**Height (cm)**
Electroacupuncture	37	49.90 ± 4.6	159.02 ± 2.9
Control	36	51.50 ± 4.4	157.35 ± 5.3

The inclusion criteria of patients in this study were as follows: (1) patients were diagnosed with perimenopausal and abdominal obesity; (2) patients had not received weight-loss treatment in the past 3 months. The exclusion criteria of patients in this study were as follows: (1) patients with secondary obesity caused by hypothalamic syndrome, pituitary tumor, Cushing’s syndrome, hypothyroidism, insulinoma, or polycystic ovary syndrome; (2) patients who were diagnosed with uterine or ovarian malformations, have undergone uterine or ovarian resection, or in pregnancy or breastfeeding; (3) patients with severe diseases such as hyperthyroidism, diabetes, psychosis, malignant tumors, heart, liver, and kidney diseases, and hematopoietic system diseases.

### Study Design

The patients were randomly divided into two groups: the Control group (*n* = 36) and the Electroacupuncture group (*n* = 37). The patients in the Control group received healthy lifestyle education, while those in the Electroacupuncture group received electroacupuncture treatment combined with dietary intervention for 8 weeks. Before and after treatment, the stool and blood of the patients were collected for subsequent analyses, and the weight, height, waist circumference and hip circumference of the patients were recorded to calculate waist-height ratio (WHtR), waist to hip ratio (WHR), and body mass index (BMI). Here, the method of measuring waist circumference recommended by the World Health Organization is adopted: the subject takes off his shoes, hat and coat, fasts, and stands upright, with feet being separated by 25–30 cm. The measurement position is at the midpoint of the line between the horizontal anterior superior iliac crest and the inferior edge of the twelfth rib.

### Electroacupuncture Treatment

The main acupuncture meridians are Renmai, Stomach, Spleen, and Bladder Meridians. The acupoints are Zhongwan, Qihai, Guanyuan, Tianshu, Daheng, Pishu, Shenshu, and Dachangshu. The acupoint combinations in the study were as follows: Sanyinjiao and Inner Court were combined for the patients who belonged to the gastric-heat stagnation type; Yinlingquan and Fenglong were combined for the patients who pertained to the phlegm dampness Neisheng type; Hegu and Taichong were combined for the patients who appeared liver stagnation; Zusanli and Taibai were combined for the patients with spleen deficiency; and Mingmen and Shenmai were combined for the patients who presented symptoms of spleen and kidney yang.

Regarding the treatment, 0.25 mm × 40 mm (190,030) and 0.30 mm × 50 mm (190,185) disposable acupuncture needles (Huatuo, Suzhou, China) as well as an SDZ-V electronic acupuncture instrument (Huatuo, Suzhou, China) were employed. 1.5 inch acupuncture needles were used at the Backshu acupoints and four-limb acupoints. For the Backshu acupoints, the needle was inserted obliquely at 45° into the spine at 1-inch depth, while for the four-limb acupoints, the needle was inserted straight at 0.8–1.0-inch depth (0.3–0.5-inch depth for Taibai, Taichong, and Neiting). 2.0 inch acupuncture needles were inserted at the acupoints on the abdomen at 2-inch depth. Electroacupuncture was applied to Tianshu, Abdomen Jie, Daheng, and Meridian, and the waveform was a dense wave with a frequency of 2/100Hz. The needle was retained for 30 min. The acupuncture acupoints without electroacupuncture treatment were slightly lifted and twisted3 times every 10 min. Patients in the Electroacupuncture group received electroacupuncture treatment every 2 days (3 times a week) for 8 weeks.

### Dietary Intervention

Patients in the Electroacupuncture group were given a strict low-sugar, low-fat, low-salt, and high-fiber diet. The diet contained staple foods, meat, and vegetables (each accounting for one third of the total dietary ingestion), fruits, and vegetables (no less than 250 g), and mineral supplements. Besides, the patients drunk 2,000–2,500 ml of water and no more than 20 g of liqueur, ingested less than 6 g of salt per day, and only ate fish products twice a week. Here, dietary intervention was implemented on the basis of menopausal health management strategies (Zhang, 2015).

### Analysis of Physical Indexes

Before and after treatment, the weight (kg), height (cm), waist circumference (cm), and hip circumference (cm) of the patients were recorded. Then WHtR, WHR, and BMI were calculated using the following formulae: WHtR = waist/height; WHR = waist/hip; BMI = weight/height^2^.

### Analysis of Serum Indexes

Before and after treatment, the serum of all the patients was analyzed using an Automatic biochemical analyzer (BS-280; mindray, Shenzhen, China) to evaluate the levels of serum triglyceride (TG), total cholesterol (TC), low-density lipoprotein (LDL), high-density lipoprotein cholesterol (HDL-C), fasting insulin (FINS), and fasting blood glucose (FBG). Besides, Homeostasis Model Assessment-insulin resistance (HOMA-IR) was calculated according to the formula: HOMA-IR = FBG × FINS/22.5.

### Analysis of Intestinal Flora

The intestinal flora in the patients was detected by 16s rRNA sequencing technology according to the previous publication (Di Segni et al., 2018). In brief, after the stool samples were collected, the RNA in the samples was extracted and subsequently subjected to polymerase chain reaction (PCR) (16s rRNA forward primer: 5′-GTGCCAGCMGCCGCGG-3′; 16s rRNA reversed primer: 5′-CCGTCAATTCMTTTRAGTTT-3′). Then the PCR product was harvested and sequenced through Illumina system. Meantime, the Clean Tags of the PCR product were obtained for the Operational Taxonomic Units (OTU) analysis.

In line with the clustering results of OTU, the representative sequence of each OTU was annotated to obtain the corresponding species annotation information and species abundance distribution. Then, the species abundance in the sample and the species differences between samples were obtained through the visual statistical analysis of OTUs, and the Venn and Partial Least Squares Discrimination Analysis (PLS-DA) diagrams were plotted. Next, the information was subjected to Alpha diversity analysis to acquire the community structure information with differences in the sample. The Linear discriminant analysis (LDA) effect size (LEfSe) statistical analysis was applied to further investigate the differences in community structure among samples, and to identify different species via testing the species composition and community results of the grouped samples.

### Statistical Analysis

All data involved in this study except for those from intestinal flora analysis were tested the normality of the variable distribution and analyzed by student’s t-test. Statistical data were presented as mean ± standard deviation. The data were recognized as statistically significant when *P* < 0.05. These analyses were performed under SPSS 20.0 software.

## Results

### Electroacupuncture Combined With Diet Treatment Ameliorated the Physical Indexes of Patients

Given that there was no statistical difference in the general information (cases, age, and height) (Table 1) of the two groups of patients, the patients in the two groups received different treatments. The physical indexes (weight, waist circumference, hip circumference, WHtR, WHR, and BMI) (Table 2) of patients in the two groups before and after treatment were documented. As shown in Table 2, in the Electroacupuncture group, the weight (*P* < 0.01), waist circumference (*P* < 0.01), hip circumference (*P* < 0.01), BMI (*P* < 0.01), WHtR (*P* < 0.01), and WHR (*P* < 0.01) of the patients were decreased after treatment when compared with those before treatment, while the patients in the Control group after treatment showed a larger waist circumference (*P* < 0.01) and a higher WHR (*P* < 0.01) than before treatment. Additionally, the waist circumference (*P* < 0.01), WHtR (*P* < 0.01), and WHR (*P* < 0.01) of patients in the Electroacupuncture group were notably lower than those in the Control group, which indicated that electroacupuncture combined with diet treatment might ameliorate abdominal obesity in perimenopausal patients.

**TABLE 2:** Comparison of physical indexes between two groups of patients before and after the treatment.

**Physical index**	**Group**	**Before**	**After**
Weight (kg)	Electroacupuncture	61.56 ± 7.90	59.64 ± 7.27^△△^
	Control	60.03 ± 6.06	60.03 ± 5.89
Waist circumference (cm)	Electroacupuncture	88.77 ± 6.47	84.81 ± 6.17^**△△^
	Control	88.22 ± 5.07	89.92 ± 5.66^△△^
Hips (cm)	Electroacupuncture	96.70 ± 5.00	93.50 ± 4.42^△△^
	Control	95.00 ± 4.88	95.36 ± 4.65
BMI (kg⋅m^–2^)	Electroacupuncture	24.33 ± 2.95	23.58 ± 2.76^△△^
	Control	24.45 ± 2.41	24.21 ± 1.91
WHtR	Electroacupuncture	0.558 ± 0.041	0.534 ± 0.040^**△△^
	Control	0.566 ± 0.029	0.567 ± 0.036
WHR	Electroacupuncture	0.918 ± 0.038	0.907 ± 0.041^**△△^
	Control	0.931 ± 0.050	0.943 ± 0.039^△△^

*^∗∗^*P* < 0.01, vs. Control; ^△△^*P* < 0.01, vs. Before. Hips, hip circumference; BMI, body mass index; WHtR, waist-height ratio; WHR, waist-to-hip ratio.*

### Electroacupuncture Combined With Diet Treatment Refined the Serum Indexes of Patients

Then the levels of TG, TC, LDL, HDL-C, FINS, FBG, and HOMA-IR of the patients in the two groups before and after treatment were evaluated. As illustrated in Table 3, in the Control group, the LDL level of patients after treatment was elevated (*P* < 0.05) as compared with that before treatment. Moreover, the TG (*P* < 0.01) and LDL levels (*P* < 0.01) of patients in the Electroacupuncture group were markedly lower than those in the Control group, which proved that electroacupuncture combined with diet treatment ameliorated the TG and LDL levels of perimenopausal patients with abdominal obesity.

**TABLE 3:** Comparison of serum indexes between two groups of patients before and after the treatment.

**Serum index**	**Group**	**Before**	**After**
TG (mmol⋅L^–1^)	Electroacupuncture	1.62 ± 1.12	1.62 ± 0.85
	Control	1.40 ± 0.49	1.61 ± 0.67
TC (mmol⋅L^–1^)	Electroacupuncture	4.97 ± 0.91	4.80 ± 0.70^∗∗^
	Control	5.14 ± 1.25	5.25 ± 0.86
LDL (mmol⋅L^–1^)	Electroacupuncture	3.05 ± 0.82	2.98 ± 0.65^∗∗^
	Control	3.16 ± 1.11	3.49 ± 0.69^△^
HDL-C (mmol⋅L^–1^)	Electroacupuncture	1.27 ± 0.17	1.28 ± 0.25
	Control	1.31 ± 0.21	1.35 ± 0.30
FINS (pmol⋅L^–1^)	Electroacupuncture	60.48 ± 33.01	56.35 ± 30.22
	Control	56.77 ± 30.85	60.55 ± 18.68
HOMA-IR	Electroacupuncture	14.63 ± 8.65	12.89 ± 6.68
	Control	16.28 ± 16.47	12.83 ± 4.77
FBG (mmol⋅L^–1^)	Electroacupuncture	5.41 ± 0.81	5.06 ± 0.55
	Control	5.09 ± 0.42	5.02 ± 0.41

*^∗∗^*P* < 0.01, vs. Control; ^△^*P* < 0.05, vs. Before. TG, serum triglyceride; TC, total cholesterol; LDL, low-density lipoprotein; HDL-C, high-density lipoprotein cholesterol; FINS, fasting insulin; FBG, fasting blood glucose; HOMA-IR, Homeostasis Model Assessment-insulin resistance.*

### Electroacupuncture Combined With Diet Treatment Regulated the OTU Number of the Intestinal Flora in Patients

**FIGURE 1:**
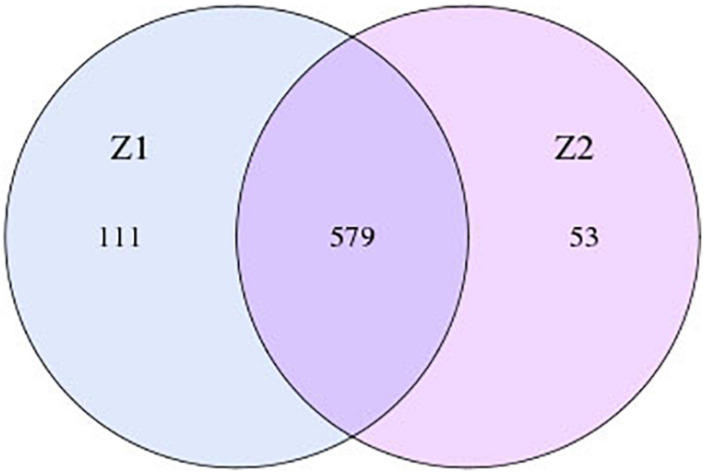
OTU numbers of intestinal flora in the patients in the Electroacupuncture group before and after treatment (Z1, the Electroacupuncture group before treatment; Z2, the Electroacupuncture group after treatment; D1, the Control group before treatment; D2: the Control group after treatment).

**FIGURE 2:**
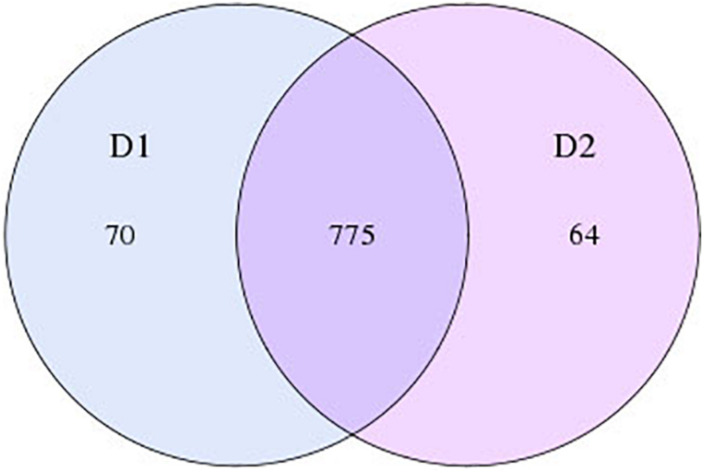
OTU numbers of intestinal flora in the patients in the Control group before and after treatment (Z1, the Electroacupuncture group before treatment; Z2, the Electroacupuncture group after treatment; D1, the Control group before treatment; D2, the Control group after treatment).

During the analysis of intestinal flora, the information in the Electroacupuncture group before and after treatment was named Z1 team and Z2 team, respectively, and the information in the Control group before and after treatment was called D1 team and D2 team, respectively. As depicted in Figure 1, in the Electroacupuncture group, the number of OTUs that existed both before and after treatment was 579, and that of specific OTUs was reduced from 111 to 53 after treatment. Meanwhile, in the Control group (Figure 2), the number of OTUs that existed both before and after treatment was 775, and that of specific OTUs was decreased to 64 after treatment. These discoveries indicated that electroacupuncture combined with diet treatment decreased the abundance of the intestinal flora in the patients.

**FIGURE 3:**
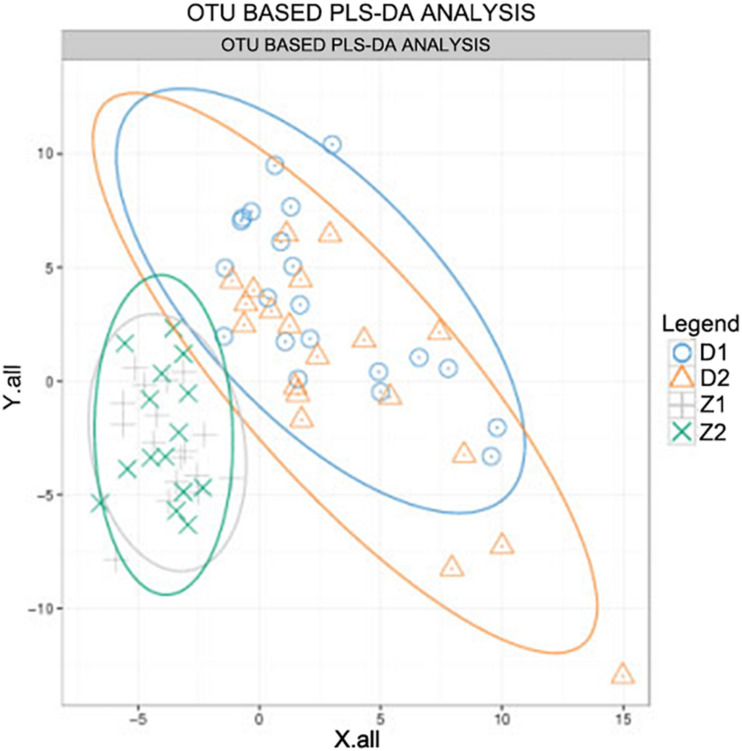
The PLS-DA analysis of OTU determination in the Electroacupuncture group and the Control group (Z1, the Electroacupuncture group before treatment; Z2, the Electroacupuncture group after treatment; D1, the Control group before treatment; D2, the Control group after treatment).

In addition, according to the PLS-DA analysis based on OTU determination (Figure 3), the samples belonging to the same group (the Electroacupuncture group or the Control group) in each team (Z1 and Z2, or D1 and D2) were almost identical to each other; the structure site of the flora sample in each team was relatively low; the distance between the samples of team Z (the Electroacupuncture group) and team D (the Control group) was far; and the difference in bacterial community structure between the two groups was relatively significant. All these results verified that the grouping results were reasonable, accompanied by satisfactory efficiency of the classification model.

### Electroacupuncture Combined With Diet Treatment Modulated the Phylum and Genus Levels of Intestinal Flora in Patients

**FIGURE 4:**
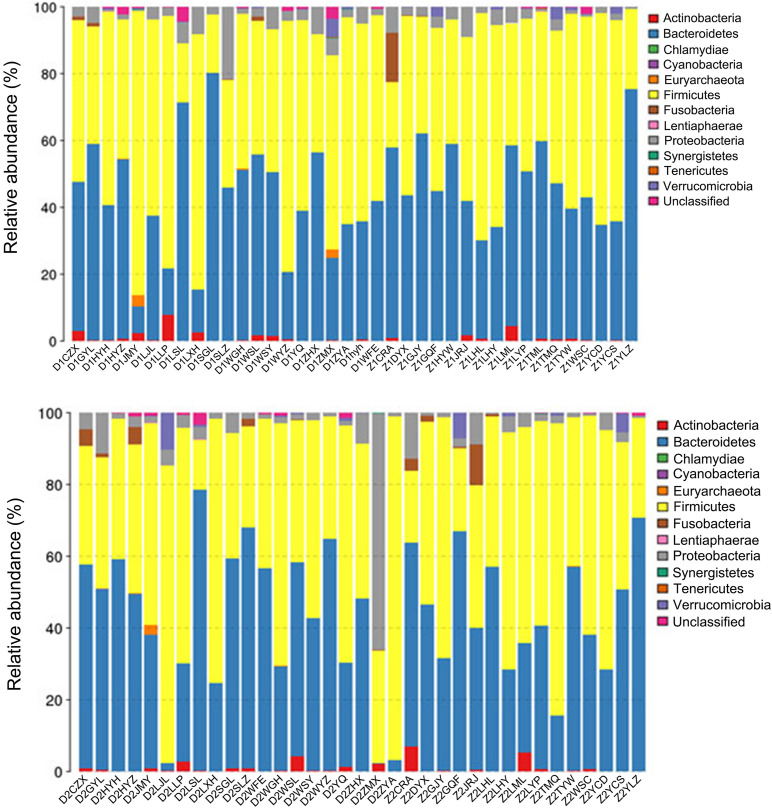
Phylum-level changes of intestinal flora in the patients in the Electroacupuncture group and the Control group (Z1, the Electroacupuncture group before treatment; Z2, the Electroacupuncture group after treatment; D1, the Control group before treatment; D2, the Control group after treatment).

**FIGURE 5:**
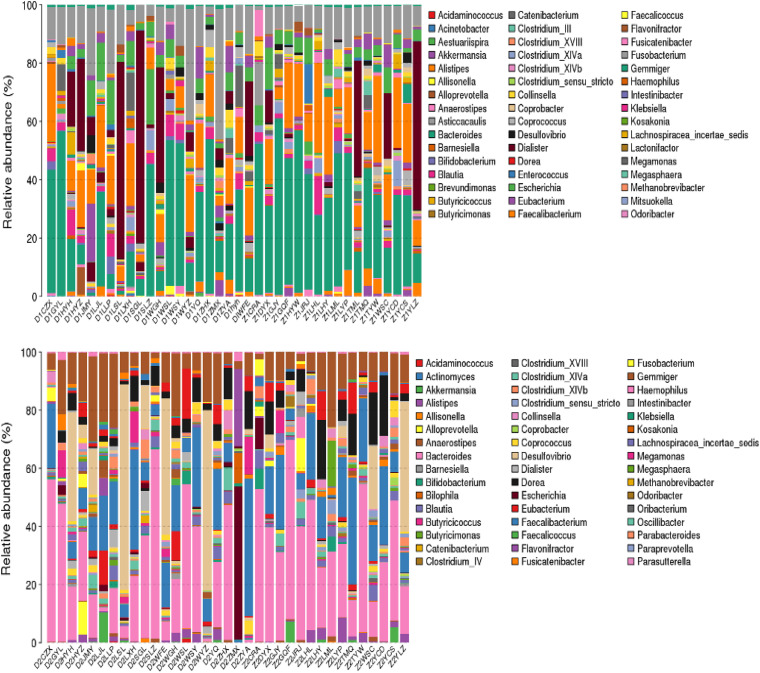
Genus-level changes of intestinal flora in the patients in the Electroacupuncture group and the Control group. (Z1, the Electroacupuncture group before treatment; Z2, the Electroacupuncture group after treatment; D1, the Control group before treatment; D2, the Control group after treatment).

The phylum-level and genus-level changes of the intestinal flora in patients before and after treatment were exhibited in Figures 4, 5, respectively. As delineated in Figure 4 and Table 4, at the phylum level, the most abundant flora in both groups before (D1 and Z1) and after (D2 and Z2) treatment were Firmicutes, Bacteroidetes, and Proteobacteria. However, difference appeared at the genus-level, as shown in Figure 5 and Table 5. Although the most dominant flora in both groups before (D1 and Z1) and after (D2 and Z2) treatment were Bacteroides, Prevotella, Faecalibacterium, and Roseburia, the Enterococcus level was decreased (*P* < 0.05) and the Enterobacter level was increased in Z2 group, when compared with the Z1 group (*P* < 0.05), while the Blautia level was lessened (*P* < 0.05) and the Enterobacter level was augmented in D2 group, when compared with the D1 group (*P* < 0.05).

**TABLE 4:** Comparison of Phylum level between two groups of patients before and after treatment (x ± s).

**Phylum levels**	**Groups**	**Before**	**After**
Firmicutes	Electroacupuncture	49.12 ± 11.58	55.76 ± 15.76
	Control	50.16 ± 18.44	47.72 ± 21.38
Bacteroidetes	Electroacupuncture	47.64 ± 12.24	44.29 ± 15.51
	Control	39.85 ± 20.12	42.02 ± 22.34
Proteobacteria	Electroacupuncture	3.12 ± 2.36	3.60 ± 3.53
	Control	5.45 ± 6.42	6.42 ± 14.70

**TABLE 5:** Comparison of genus level between two groups of patients before and after treatment (x ± s).

**Genus levels**	**Groups**	**Before**	**After**
Bacteroides	Electroacupuncture	35.06 ± 14.17	34.71 ± 15.15
	Control	22.99 ± 19.18	24.63 ± 20.25
Prevotella	Electroacupuncture	7.74 ± 16.81	4.65 ± 11.86
	Control	13.14 ± 22.24	13.35 ± 22.43
Faecalibacterium	Electroacupuncture	15.66 ± 9.04	14.18 ± 10.63
	Control	11.79 ± 7.38	13.35 ± 12.35
Roseburia	Electroacupuncture	4.57 ± 3.43	7.61 ± 7.23
	Control	4.70 ± 4.14	3.76 ± 3.43
Blautia	Electroacupuncture	2.51 ± 2.98	2.26 ± 1.75
	Control	2.98 ± 2.60	1.41 ± 1.52^Δ^
Enterobacter	Electroacupuncture	0.16 ± 0.24	0.17 ± 0.50^Δ^
	Control	0.33 ± 0.55	0.75 ± 2.67^Δ^
Enterobacter	Electroacupuncture	0.005 ± 0.01	0.0001 ± 0.0005^Δ^
	Control	0.18 ± 0.70	0.02 ± 0.08

*^Δ^P < 0.05, vs. Before.*

### Electroacupuncture Combined With Diet Treatment Regulated the Alpha Diversity of Intestinal Flora in Patients

**FIGURE 6:**
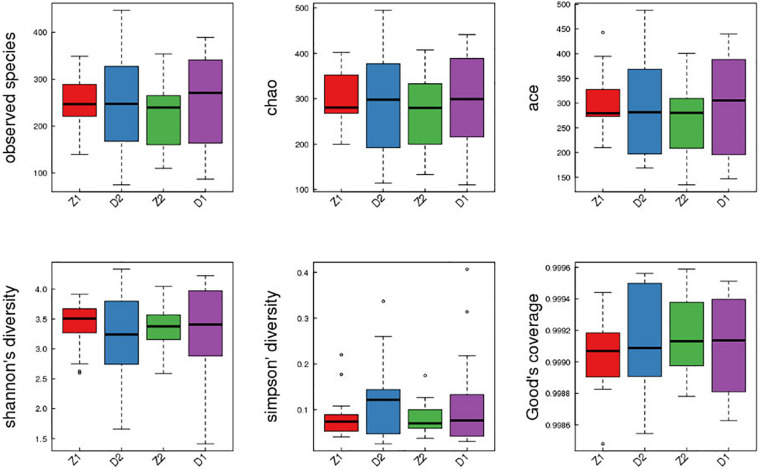
Alpha diversity of intestinal flora in the patients in the Electroacupuncture group and the Control group (Z1, the Electroacupuncture group before treatment; Z2, the Electroacupuncture group after treatment; D1, the Control group before treatment; D2, the Control group after treatment).

The Alpha diversity of intestinal flora was evaluated. As shown in Figure 6 and Table 6, although there was no statistically difference between Z1 and Z2 groups, the observed species, chao, ace, and shannon indexes in the Electroacupuncture group after treatment (Z2) showed an upward trend, while the simpson index presented a downward trend, signifying that the species abundance in the Electroacupuncture group after treatment was on the rise. Meanwhile, despite no statistically difference between D1 and D2 groups, the same observed indexes as above in D2 group showed a downward trend, while the simpson index was on the contrary, manifesting that the species abundance in the Control group was inhibited. These results indicated that electroacupuncture combined with diet treatment improved the species diversity of intestinal flora in patients.

**TABLE 6:** Comparison of Alpha diversity between two groups of patients before and after treatment (x ± s).

**Genus levels**	**Groups**	**Before**	**After**
Observed species	Electroacupuncture	221.19 ± 68.04	251.77 ± 92.71
	Control	247.18 ± 55.98	246.85 ± 95.28
Chao	Electroacupuncture	271.37 ± 81.44	295.04 ± 94.56
	Control	301.97 ± 65.14	286.79 ± 102.80
Ace	Electroacupuncture	266.67 ± 70.47	295.53 ± 94.38
	Control	302.49 ± 61.10	290.10 ± 95.63
Shannon	Electroacupuncture	3.33 ± 0.38	3.51 ± 0.74
	Control	3.40 ± 0.42	3.21 ± 0.74
Simpson	Electroacupuncture	0.08 ± 0.03	0.07 ± 0.10
	Control	0.08 ± 0.05	0.17 ± 0.08

### Electroacupuncture Combined With Diet Treatment Regulated the Species Differences of Intestinal Flora in Patients

**FIGURE 7:**
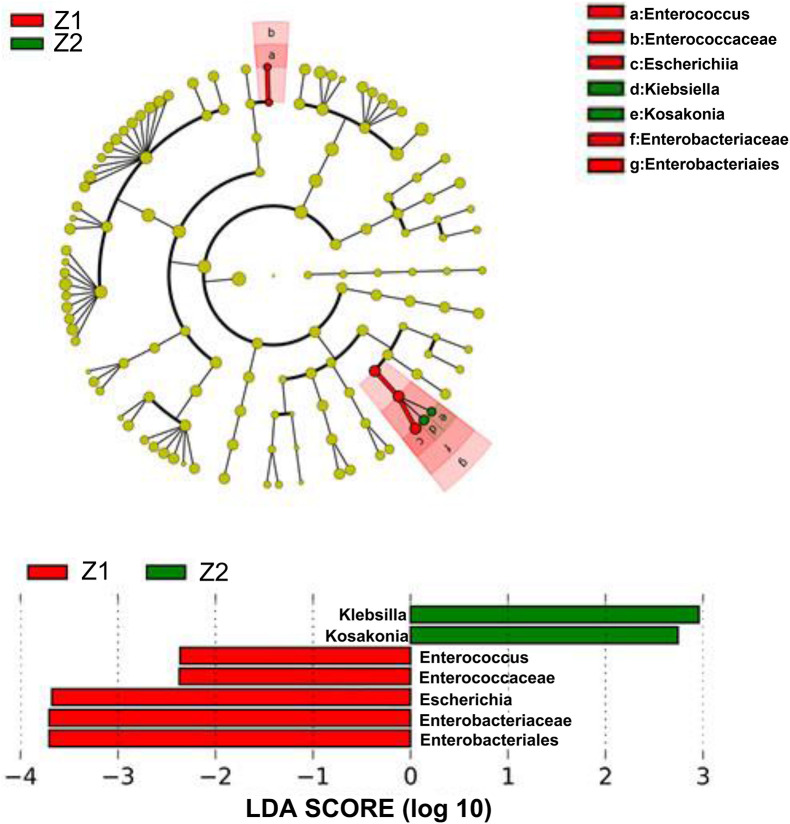
Species differences of intestinal flora in the patients in the Electroacupuncture group and the Control group (Z1, the Electroacupuncture group before treatment; Z2, the Electroacupuncture group after treatment; D1, the Control group before treatment; D2, the Control group after treatment).

The species difference of intestinal flora in the Electroacupuncture group was analyzed. As profiled in Figure 7, the abundance of Enterococcus, Enterococcaceae, Escherichia, Enterobacteriaceae, and Enterobacteriales was reduced yet that of Klebsiella and Kosakonia was enhanced after treatment, which demonstrated that electroacupuncture combined with diet treatment increased the species differences of intestinal flora in patients, and Klebsiella and Kosakonia were instrumental in the treatment of abdominal obesity in perimenopausal patients.

## Discussion

More and more researches have proved that acupuncture can safely and effectively treat abdominal obesity, mainly by stimulating the body’s meridians and acupoints, improving body condition as a whole, regulating intake, and enhancing body metabolism (Zhang et al., 2019). Acupuncture meridians such as the spleen, stomach, liver, and kidney can warm the kidney and strengthen the spleen, soothe the liver and regulate the qi, adjust the function of the internal organs, enhance metabolism, smooth the blood flow, and invigorate the blood of the five internal organs, thereby fulfilling the purpose of weight loss and waist thinning (Wang et al., 2008; Lei et al., 2017; Chen et al., 2018; Luo et al., 2018; Huang et al., 2019; Zhang et al., 2019). In this study, to confirm the effect of electroacupuncture combined with dietary intervention on abdominal obesity, the physical indexes of patients were first detected after treatment. We discovered that electroacupuncture combined with diet treatment reduced the weight, waist circumference, hip circumference, BMI, WHtR, and WHR of the patients when compared with those before treatment, and besides, the combined treatment of electroacupuncture and diet reduced the waist circumference, WHtR, and WHR of patients as compared to patients receiving healthy lifestyle education, which connoted that electroacupuncture combined with diet treatment was feasible in ameliorating abdominal obesity in perimenopausal patients.

In addition to the physical indexes, there are also a series of serum indexes which are closely related to the glycolipid metabolism of obesity patients. TG and TC are two important factors in the glycolipid metabolism, which both display higher levels in obesity patients than in healthy people (Wiesner and Watson, 2017). Furthermore, LDL, a small-diameter lipoprotein, is the main transfer tool in the process of cholesterol transport. Abnormal LDL level is indicative of dyslipidemia, one of the symptoms of obese patients (Howard et al., 2003; Chan et al., 2004; Klop et al., 2013). A study found that electroacupuncture reduced the serum TC, TG, and LDL levels in obese women (Cabioglu and Ergene, 2005), and the same downtrend was discovered in this study when applied electroacupuncture combined with diet treatment to patients, which further verified that electroacupuncture combined with diet treatment had the ability to alleviate abdominal obesity in perimenopausal patients through regulating the serum levels of TG, TC, and LDL.

As one of the complex ecosystems in the gastrointestinal tract, the intestinal flora is related to metabolism-related diseases and multi-system diseases (Di Segni et al., 2018). Acupuncture was proved to alleviate obesity-related lipid metabolism disorders and intestinal microbiota disorders (Gao et al., 2020; Xie et al., 2020). For example, electroacupuncture promoted the diversity of the gut microbiota of obese mice, thus reducing body weight (Dou et al., 2020). Si et al. (2018) found that the structure of the intestinal flora of animals in the acupuncture group was gradually similar to those in the healthy control group. Therefore, we then focused on investigating whether the effect of electroacupuncture on abdominal obesity was realized by regulating the community structure of the intestinal flora in the patients.

The high-throughput sequencing technology has been widely used in microbiome analysis, because it allows scholars to sequence millions of DNA molecules simultaneously and provides a data pool to cover the entire microbiome in the gut (Gao X. et al., 2018). On the flip side, the gene encoding 16s rRNA is the most commonly used target for studying bacterial evolution and classification (Gao X. et al., 2018; Li et al., 2020). Hence, in the present study, we used 16s rRNA sequencing technology to analyze the information of the intestinal flora in the patients before and after treatment. The previous study has discovered higher species abundance, lower species diversity, and smaller species differences of the intestinal flora in obese patients (Malamut, 2007; Yazigi et al., 2008; Gao X. et al., 2018; Li et al., 2020). Based on the OTU corresponding species annotation, Alpha diversity, and LEfSe analyses, we for the first time unveiled that electroacupuncture combined with diet treatment lessened the species abundance, and increased the species diversity and differences of intestinal flora in perimenopausal patients with abdominal obesity. Most notably, after electroacupuncture combined with diet treatment, Klebsiella and Kosakonia in patients were increased. Recent research has reported that during the antibiotic treatment of obesity mice, Klebsiella is increased to inhibit the body weight gain (Yao et al., 2020). Therefore, in this research, the role of electroacupuncture combined with diet treatment in perimenopausal patients with abdominal obesity might be realized by improving the community structure of intestinal flora and increasing Klebsiella and Kosakonia.

Taken together, electroacupuncture combined with diet treatment exerted a therapeutic effect on perimenopausal patients with abdominal obesity through improving the community structure of intestinal flora. Nevertheless, what failed to be underscored in our study is the design of separate dietary intervention group, that is, the same diet restriction was performed without electroacupuncture, which may be further explored in future studies. Consumption patterns may affect obesity, which may be another aspect that we need to pay attention to in future research.

## Data Availability Statement

The raw data supporting the conclusions of this article will be made available by the authors, without undue reservation.

## Ethics Statement

The study has been approved by the Ethics Committee of Zhejiang Hospital [No. 2018 (9K)] and all patients signed the written informed consent.

## Author Contributions

JS and GY: substantial contributions to conception and design and drafting the article or critically revising it for important intellectual content. JZ, CS, YH, ZL, TL, and XJ: data acquisition, data analysis and interpretation. JS, GY, JZ, CS, Y’aH, ZL, TL, and XJ: final approval of the version to be published and agreement to be accountable for all aspects of the work in ensuring that questions related to the accuracy or integrity of the work are appropriately investigated and resolved. All authors contributed to the article and approved the submitted version.

## Conflict of Interest

The authors declare that the research was conducted in the absence of any commercial or financial relationships that could be construed as a potential conflict of interest.

## Publisher’s Note

All claims expressed in this article are solely those of the authors and do not necessarily represent those of their affiliated organizations, or those of the publisher, the editors and the reviewers. Any product that may be evaluated in this article, or claim that may be made by its manufacturer, is not guaranteed or endorsed by the publisher.

## References

[B1] Bronczyk-Puzon A. Piecha D. Nowak J. Koszowska A. Kulik-Kupka K. Dittfeld A. (2015). Guidelines for dietary management of menopausal women with simple obesity. *Prz. Menopauzalny* 14 48–52. 10.5114/pm.2015.48678 26327888 PMC4440197

[B2] Cabioglu M. T. Ergene N. (2005). Electroacupuncture therapy for weight loss reduces serum total cholesterol, triglycerides, and LDL cholesterol levels in obese women. *Am. J. Chin. Med.* 33 525–533. 10.1142/S0192415X05003132 16173527

[B3] Chan D. C. Barrett H. P. Watts G. F. (2004). Dyslipidemia in visceral obesity: mechanisms, implications, and therapy. *Am. J. Cardiovasc. Drugs* 4 227–246. 10.2165/00129784-200404040-00004 15285698

[B4] Chen X. Huang W. Jin Y. Hu F. Cheng X. Hong Z. (2018). [Prescription analysis of electroacupuncture for simple obesity based on complex network technique]. *Zhongguo zhen jiu* 38 331–336. 10.13703/j.0255-2930.2018.03.028 29701055

[B5] Di Segni A. Braun T. BenShoshan M. Farage Barhom S. Glick Saar E. Cesarkas K. (2018). Guided protocol for fecal microbial characterization by 16S rRNA-amplicon sequencing. *J. Vis. Exp.* 133:56845. 10.3791/56845 29608151 PMC5933208

[B6] Dou D. Chen Q. Q. Zhong Z. Q. Xia X. W. Ding W. J. (2020). Regulating the enteric nervous system against obesity in mice by electroacupuncture. *Neuroimmunomodulation* 27 48–57. 10.1159/000506483 32516787

[B7] Gao X. Jia R. Xie L. Kuang L. Feng L. Wan C. (2018). A study of the correlation between obesity and intestinal flora in school-age children. *Sci. Rep.* 8:14511. 10.1038/s41598-018-32730-6 30267022 PMC6162261

[B8] Gao Y. Bi W. Wu X. Zhu X. Luo Y. (2018). [Bacterial resistance influences intestinal flora and host immune regulation]. *Sheng wu gong cheng xue* 34 1259–1269. 10.13345/j.cjb.180123 30152211

[B9] Gao Y. Wang Y. Zhou J. Hu Z. Shi Y. (2020). Effectiveness of electroacupuncture for simple obesity: a systematic review and meta-analysis of randomized controlled trials. *Evid. Based Complement. Alternat. Med.* 2020:2367610. 10.1155/2020/2367610 32714399 PMC7341404

[B10] Gazarova M. Galsneiderova M. Meciarova L. (2019). Obesity diagnosis and mortality risk based on a body shape index (ABSI) and other indices and anthropometric parameters in university students. *Rocz. Panstw. Zakl. Hig.* 70 267–275. 10.32394/rpzh.2019.0077 31515986

[B11] González-Muniesa P. Mártinez-González M. A. Hu F. B. Després J. P. Matsuzawa Y. Loos R. J. F. (2017). Obesity. *Nat. Rev. Dis. Primers* 3:17034. 10.1038/nrdp.2017.34 28617414

[B12] Howard B. V. Ruotolo G. Robbins D. C. (2003). Obesity and dyslipidemia. *Endocrinol. Metab. Clin. North Am.* 32 855–867. 10.1016/s0889-8529(03)00073-2 14711065

[B13] Hu L. Huang X. You C. Li J. Hong K. Li P. (2017). Prevalence of overweight, obesity, abdominal obesity and obesity-related risk factors in southern China. *PLoS One* 12:e0183934. 10.1371/journal.pone.0183934 28910301 PMC5598943

[B14] Huang Q. Chen R. Chen L. Liang F. X. He W. J. Peng M. (2019). [Electroacupuncture reduces obesity by improving metabolism and up-regulating expression of hypothalamic Sirtuin 1 and proopiomelanocortin in obese rats]. *Zhen ci yan jiu = Acupuncture research* 44 270–275. 10.13702/j.1000-0607.180190 31056880

[B15] Jablonowska-Lietz B. Wrzosek M. Wlodarczyk M. Nowicka G. (2017). New indexes of body fat distribution, visceral adiposity index, body adiposity index, waist-to-height ratio, and metabolic disturbances in the obese. *Kardiol. Pol.* 75 1185–1191. 10.5603/KP.a2017.0149 28715064

[B16] Keller C. Larkey L. Distefano J. K. Boehm-Smith E. Records K. Robillard A. (2010). Perimenopausal obesity. *J. Womens Health (Larchmt)* 19 987–996. 10.1089/jwh.2009.1547 20380578

[B17] Klop B. Elte J. W. Cabezas M. C. (2013). Dyslipidemia in obesity: mechanisms and potential targets. *Nutrients* 5 1218–1240. 10.3390/nu5041218 23584084 PMC3705344

[B18] Lei H. Chen X. Liu S. Chen Z. (2017). Effect of electroacupuncture on visceral and hepatic fat in women with abdominal obesity: a randomized controlled study based on magnetic resonance imaging. *J. Altern. Complement. Med. (New York, NY)* 23 285–294. 10.1089/acm.2016.0361 28394670

[B19] Li X. Shi W. Xiong Q. Hu Y. Qin X. Wan G. (2020). Leptin improves intestinal flora dysfunction in mice with high-fat diet-induced obesity. *J. Int. Med. Res.* 48:300060520920062. 10.1177/0300060520920062 32529880 PMC7294385

[B20] Łokieć K. Błońska A. Walecka-Kapica E. Stec-Michalska K. (2016). [Effect of treatment with diet on reducing levels of sex hormones in perimenopausal women with overweight and obesity]. *Pol. Merkur. Lekarski* 40 362–368. 27403902

[B21] Luo D. Liu L. Liang F. X. Yu Z. M. Chen R. (2018). Electroacupuncture: a feasible sirt1 promoter which modulates metainflammation in diet-induced obesity rats. *Evid. Based Complement. Alternat. Med.* 2018:5302049. 10.1155/2018/5302049 30425749 PMC6217753

[B22] Ma M. J. Wu J. (2017). [Association between intestinal flora imbalance and nonalcoholic fatty liver disease]. *Zhonghua Gan Zang Bing Za Zhi* 25 789–793. 10.3760/cma.j.issn.1007-3418.2017.10.017 29108214 PMC12769113

[B23] Malamut G. (2007). [Obesity and intestinal flora]. *Gastroenterol. Clin. Biol.* 31 (8-9 Pt 1) 761–762. 10.1016/s0399-8320(07)91941-7 17925783

[B24] Mastorakos G. Valsamakis G. Paltoglou G. Creatsas G. (2010). Management of obesity in menopause: diet, exercise, pharmacotherapy and bariatric surgery. *Maturitas* 65 219–224. 10.1016/j.maturitas.2009.12.003 20044222

[B25] Mohammadbeigi A. Asgarian A. Moshir E. Heidari H. Afrashteh S. Khazaei S. (2018). Fast food consumption and overweight/obesity prevalence in students and its association with general and abdominal obesity. *J. Prev. Med. Hyg.* 59 E236–E240. 10.15167/2421-4248/jpmh2018.59.3.830 30397681 PMC6196377

[B26] Ruban A. Doshi A. Lam E. Teare J. P. (2019). Medical devices in obesity treatment. *Curr. Diab. Rep.* 19:90. 10.1007/s11892-019-1217-3 31471810 PMC6719326

[B27] Samat A. Rahim A. Barnett A. (2008). Pharmacotherapy for obesity in menopausal women. *Menopause Int.* 14 57–62. 10.1258/mi.2008.008005 18519266

[B28] Sebastián Domingo J. J. Sánchez Sánchez C. (2018). From the intestinal flora to the microbiome. *Rev. Esp. Enferm. Dig.* 110 51–56. 10.17235/reed.2017.4947/2017 29271225

[B29] Si Y. C. Miao W. N. He J. Y. Chen L. Wang Y. L. Ding W. J. (2018). Regulating gut flora dysbiosis in obese mice by electroacupuncture. *Am. J. Chin. Med.* 46 1–17. 10.1142/S0192415X18500763 30284469

[B30] Smith U. (2015). Abdominal obesity: a marker of ectopic fat accumulation. *J. Clin. Investig.* 125 1790–1792. 25932676 10.1172/JCI81507 PMC4463217

[B31] Tauqeer Z. Gomez G. Stanford F. C. (2018). Obesity in women: insights for the clinician. *J. Womens Health (Larchmt)* 27 444–457. 10.1089/jwh.2016.6196 29077514 PMC6110123

[B32] Vandevijvere S. Chow C. C. Hall K. D. Umali E. Swinburn B. A. (2015). Increased food energy supply as a major driver of the obesity epidemic: a global analysis. *Bull. World Health Organ.* 93 446–456. 10.2471/BLT.14.150565 26170502 PMC4490816

[B33] Wang F. Tian D. R. Han J. S. (2008). Electroacupuncture in the treatment of obesity. *Neurochem. Res.* 33 2023–2027. 10.1007/s11064-008-9822-6 18719995

[B34] Wiesner P. Watson K. E. (2017). Triglycerides: a reappraisal. *Trends Cardiovasc. Med.* 27 428–432. 10.1016/j.tcm.2017.03.004 28438398

[B35] Xie L. L. Zhao Y. L. Yang J. Cheng H. Zhong Z. D. Liu Y. R. (2020). Electroacupuncture prevents osteoarthritis of high-fat diet-induced obese rats. *Biomed. Res. Int.* 2020:9380965. 10.1155/2020/9380965 32724821 PMC7366230

[B36] Yao H. Fan C. Lu Y. Fan X. Xia L. Li P. (2020). Alteration of gut microbiota affects expression of adiponectin and resistin through modifying DNA methylation in high-fat diet-induced obese mice. *Genes Nutr.* 15:12. 10.1186/s12263-020-00671-3 32586265 PMC7318443

[B37] Yazigi A. Gaborit B. Nogueira J. P. Butiler M. E. Andreelli F. (2008). [Role of intestinal flora in insulin resistance and obesity]. *Presse Med. (Paris, France : 1983)* 37 1427–1430. 10.1016/j.lpm.2007.11.020 18450416

[B38] Zhang S. F. B. L. (2015). Menopausal health management strategies. *J. Pract. Obstet. Gynecol.* 31 333–334.

[B39] Zhang Y. J. Li J. Huang W. Mo G. Y. Wang L. H. Zhuo Y. (2019). [Effect of electroacupuncture combined with treadmill exercise on body weight and expression of PGC-1α, Irisin and AMPK in skeletal muscle of diet-induced obesity rats]. *Zhen ci yan jiu* 44 476–480. 10.13702/j.1000-0607.180460 31368276

