# Corrigendum: Electroacupuncture Combined With Diet Treatment Has a Therapeutic Effect on Perimenopausal Patients With Abdominal Obesity by Improving the Community Structure of Intestinal Flora

**DOI:** 10.3389/fphys.2022.844424

**Published:** 2022-03-08

**Authors:** Jili Sheng, Geyao Yang, Xiaoqing Jin, Caijuan Si, Yuan'an Huang, Zhouxiao Luo, Tao Liu, Jianfang Zhu

**Affiliations:** ^1^Acupuncture Department, Zhejiang Hospital, Hangzhou, China; ^2^Acupuncture and Massage Department, Hangzhou Geriatric Hospital, Hangzhou, China; ^3^Nutritional Department, Zhejiang Hospital, Hangzhou, China; ^4^Massage Department, Zhejiang Hospital, Hangzhou, China; ^5^Acupuncture Department, Tonglu TCM Hospital, Hangzhou, China

**Keywords:** abdominal obesity, perimenopausal syndrome, 16S rRNA, intestinal flora, electroacupuncture

In the original article, there was an error in the **Ethics Statement** as published. A correction has been made to **Materials and Methods** subsection **Ethics Statement** and to the **Ethics Statement**:

“The study has been approved by the Ethics Committee of Zhejiang Hospital [No. 2018 (9K)] and all patients signed the written informed consent.”

The authors apologize for this error and state that this does not change the scientific conclusions of the article in any way. The original article has been updated.

## Publisher's Note

All claims expressed in this article are solely those of the authors and do not necessarily represent those of their affiliated organizations, or those of the publisher, the editors and the reviewers. Any product that may be evaluated in this article, or claim that may be made by its manufacturer, is not guaranteed or endorsed by the publisher.

