# Supplementary material for: Electroacupuncture Combined With Diet Treatment Has a Therapeutic Effect on Perimenopausal Patients With Abdominal Obesity by Improving the Community Structure of Intestinal Flora
Source: Front Physiol. 2021 Nov 25;12:708588. doi: 10.3389/fphys.2021.708588 (PMC8656264; doi:10.3389/fphys.2021.708588)
Supplement: Supplementary file 1 [file Table_1.docx]

Supplementary materials Table 1 Social demographic profile and clinical history

|  |  |  | | **Number (%)** |
| --- | --- | --- | --- | --- |
| **Age (years)** |  |  |  |  |
| 40-46 |  |  |  | 8(9.76) |
| 47-53 |  |  |  | 49(59.76) |
| 54-60 |  |  |  | 25(30.49) |
| **nationality** |  |  |  |  |
| Han nationality |  |  |  | 82(100) |
| Others |  |  |  | 0(0) |
| **Education level** |  |  |  |  |
| Illiteracy |  |  |  | 18(21.95) |
| Literacy |  |  |  | 9(10.98) |
| Primary school |  |  |  | 45(54.88) |
| Higher education |  |  |  | 10(12.2) |
| **Smoking** |  |  |  |  |
| Yes |  |  |  | 0(0.00) |
| No |  |  |  | 82(100.00) |
| **Alcoholism** |  |  |  |  |
| Yes |  |  |  | 10(12.2) |
| No |  |  |  | 72(87.8) |
| **Marital status** |  |  |  |  |
| Single |  |  |  | 7(8.54) |
| Married |  |  |  | 56(68.29) |
| Separated/divorced |  |  |  | 12(14.63) |
| Widowed |  |  |  | 7(8.54) |
| **Employment status** |  |  |  |  |
| Employed |  |  |  | 39(47.56) |
| Unemployed |  |  |  | 9(10.98) |
| Retired |  |  |  | 12(14.63) |
| Housewife |  |  |  | 22(26.83) |
